# Functionalised Fibres as a Coupling Reinforcement Agent in Recycled Polymer Composites

**DOI:** 10.3390/ma17112739

**Published:** 2024-06-04

**Authors:** Klementina Pušnik Črešnar, Olivija Plohl, Lidija Fras Zemljič

**Affiliations:** 1 Faculty of Mechanical Engineering, University of Maribor, 2000 Maribor, Slovenia; olivija.plohl@um.si (O.P.); lidija.fras@um.si (L.F.Z.); 2 Faculty of Chemistry and Chemical Engineering, University of Maribor, 2000 Maribor, Slovenia

**Keywords:** functionalised wood fibres, nanocellulose, recycled polypropylene structure properties, crystallisation, mechanical properties

## Abstract

This study addresses the structure–property relationship within the green concept of wood fibres with cellulose nanofibre functionalised composites (nW-PPr) containing recycled plastic polyolefins, in particular, polypropylene (PP-r). It focuses especially on the challenges posed by nanoscience in relation to wood fibres (WF) and explores possible changes in the thermal properties, crystallinity, morphology, and mechanical properties. In a two-step methodology, wood fibres (50% wt%) were first functionalised with nanocellulose (nC; 1–9 wt%) and then, secondly, processed into composites using an extrusion process. The surface modification of nC improves its compatibility with the polymer matrix, resulting in improved adhesion, mechanical properties, and inherent biodegradability. The effects of the functionalised WF on the recycled polymer composites were investigated systematically and included analyses of the structure, crystallisation, morphology, and surface properties, as well as thermal and mechanical properties. Using a comprehensive range of techniques, including X-ray diffraction (XRD), attenuated total reflectance Fourier transform infrared spectroscopy (ATR-FTIR), differential scanning calorimetry (DSC), thermogravimetric analysis (TGA), scanning electron microscopy (SEM), zeta potential measurements, and dynamic mechanical analysis (DMA), this study aims to unravel the intricate interplay of factors affecting the performance and properties of the developed nanocellulose-functionalised wood fibre–polymer composites. The interfacial adhesion of the nW-PPr polymer composites, crystallisation process, and surface properties was improved due to the formation of an H-bond between the nW coupling agent and neat PP-r. In addition, the role of nW (1.0 wt%) as a nucleating agent resulted in increased crystallinity, or, on the other hand, promoted the interfacial interaction with the highest amount (3.0% wt%, 9.0% wt%) of nW in the PP-r preferentially between the nW and neat PP-r, and also postponed the crystallisation temperature. The changes in the isoelectric point of the nW-PPr polymer composites compared to the neat PP-r polymer indicate the acid content of the polymer composite and, consequently, the final surface morphology. Finally, the higher storage modulus of the composites compared to neat r-PP shows a dependence on improved crystallinity, morphology, and adhesion. It was clear that the results of this study contribute to a better understanding of sustainable materials and can drive the development of environmentally friendly composites applied in packaging.

## 1. Introduction

The vision of the Plastic Europe strategy and the European Green Deal efforts is based on a sustainable, low-carbon, resource-efficient and competitive economy, fully respecting reuse, recycling, and durability [1,2,3,4]. It promotes two concepts. First, to increase the high-performance quality of recycled material, accelerate the renewable raw material and renewable energy production path technologies, addressing the nontoxic chemical with the generation of minimised waste, and second, to improve the green way of production of plastic material [3,5,6]. In line with the increasing environmental awareness, the use of functionalised wood fibres (WF) reinforced recycled plastic composites based on polyolefin polymers (PE, PET, PP, PVC) [1,3,7,8,9,10,11,12,13,14,15,16,17] offers an important opportunity to utilise raw material resources and to create high added value composites that represent a social solution, saving resources and emissions, and, ultimately, making the recycled product as attractive and as good as a neat plastic composite. The functionalisation of either the recycled polymer matrix or the fibres with coupling agents affects the improved interactions between the filler and thermoplastic polymer, provides better adhesion, and determines the final properties of such a composite. The structural changes in the recycled polymer properties (molecular weight, chain scission, crystal structure, and (trans) crystallisation behaviour), thermal properties (melting temperature and crystallisation), and rheological, mechanical, and surface behaviour of polymer composites could be comparable to nonrecycled polymer composites [18,19,20,21,22,23]. Furthermore, many coupling agents classified into organic and inorganic, including isocyanates, anhydrides, amides, amines, isocyanates, imides, acrylates, chlorotriazines, epoxides, organic acids (etc.) [21,24,25], have been used to improve the affinity and interfacial interaction between the polymer and WF filler through covalent bonding, polymer chain entanglement, and strong secondary interaction, as in the case of hydrogen bonding. However, none of these review studies address functionalised WF as a coupling agent in recycled polymer used in this field systematically, as was carried out in this study. 

The main problems of additional coupling agents are related to higher cost, can be environmentally problematic and can cause increased carbon emission [26,27,28]. In terms of sustainability and “green produced” composites, our study addresses the development of recycled material composites possibilities of new wood modification concept at the nanoscale with controllable structure, even though WF have already been used as reinforcement. They have recently become industrial practice and attracted especially the interest of the scientific community as well as a lot of attention to a wide range of applications such as automotive, aerospace, packaging, construction, and transportation [10,13,14,16,29,30,31,32,33]. Importantly, the functionalisation of WF with nanofibres is even more focused on the advantages of nanotechnologies, which provide a more effective surface area and, therefore, more available functional groups for additional interaction. 

Cellulose in the form of nanofibres, which serve as a functionalising filler, has attracted considerable attention for various applications, especially as a component of polymer composites. Nanocellulose (nC), which is derived from cellulose, exhibits unique properties in various forms that make it an attractive candidate for the modification of components such as wood fibres used as fillers in polymer composites [34,35,36]. In this context, several relevant considerations arise, including the following properties: nC, when incorporated functionally, acts as a reinforcing agent for polymeric materials, thereby improving mechanical properties [37]. A notable advantage of nC lies in its inherent biodegradability. In combination with biodegradable materials, nC contributes significantly to the development of environmentally friendly composites [38]. nC shows compatibility with various processing techniques, such as extrusion. This property increases its versatility in various manufacturing processes and enables the integration of nC in a range of composite applications [39]. Surface modification of nC proves to be a crucial strategy to improve its compatibility with polymer matrices. This modification facilitates improved dispersion and adhesion within the polymer matrix, thereby impacting the overall performance of the composite positively [37], which was confirmed by the results of the present study. Despite the benefits associated with nC-modified materials, there are still some challenges. Achieving a uniform dispersion of nC-modified fillers in the polymer matrix is a major one. Additionally, there is still a significant research gap in this area that requires further investigation to address the existing challenges comprehensively and unlock the full potential of nC in the form of fibres in polymer composites.

Generally, many reports were based on finding the influence of WF on recycled plastic properties. They reported varying lower strength and stiffness properties in HDPE, increased modulus of elasticity and tensile strength in PP and in fibreboards with HDPE [40], enhanced tensile strength and increased mechanical properties in HDPE, PP, PS, and PVC, altered hygroscopic properties compared to neat PP and HDPE polymer, a lower value of impact strength of recycled plastic compared to neat polymer [1,10,14,24,41,42,43]. In addition, functionalisation methods of WFs prepared by dispersion in water with inorganic nanoparticles, magnetic nanoparticles, carbonising strategies by nanoparticle infiltration applied for wood preservation against microorganisms and UV degradation, for fire retardant application for electromagnetic shielding, and enhanced electrical properties for energy storage application and water treatment have been used as well [44,45].

Following this, on the other hand, studies offering a detailed explanation of the mechanism of interaction interfacial phenomena, structural, thermal and mechanical properties, as well as using recycled plastic and functionalised WF with nanocellulose (nW-PPr) were not found, which represents the novelty of our research. If recycled composites reinforced with functionalised WF are to be recognised as new materials in the production of such composites, it is necessary to understand the effect of the addition of functionalised WF on the structure and the relationship between the fibres and polymer well. Moreover, in this way, all the processes for the manufacturing of recycled composites could be tuned and controlled well. The subject of our study is based on the fundamental knowledge of how the reinforcement of (nano) functionalised WF in recycled plastic matrix depends on the crystal structure of the composites and mechanical properties. Therefore, the development focuses on the modification/functionalisation of wood fibre nanoparticles and green modification by chemical and physical means to extend the range of WF composite properties to provide changes in the structure of composites, improve mechanical performance and reduce the material needed. In this way, functionalisation of nW-PPr varying biobased, recycled, nanoparticles technology leading to reduced overall environmental impact depends on the nanostructure processing and structure–property relationship. Although researchers investigated the influence of nW-PPr on recycled composites’ properties [31,43,46,47,48,49,50,51,52], no study was found on how nanocellulose functionalised WF affects recycled composites.

In view of this, the perspective of our study is the first attempt to understand the structure–property relationship of green concept WF functionalised composites based on recycled plastic polyolefins, polypropylene (PP-r), along with wood nanoscience challenges that could lead to changes in thermal properties, crystallinity, morphology, and mechanical properties. A two-step approach was evaluated: first, the WF (50% wt%) were functionalised with nC, and second, the composites were prepared during the extrusion process. The effects of functionalised WF were investigated on recycled polymer composites’ structure, crystallisation, morphology, surface properties, and thermal and mechanical properties. Thus, the investigation involves the following techniques: X-ray diffractometry (XRD), attenuated total reflectance Fourier transform infrared spectroscopy (ATR-FTIR), differential scanning calorimetry (DSC), thermogravimetric analysis (TGA), scanning electron microscopy (SEM), zeta potential measurements, and the technique of dynamic mechanical analysis (DMA). 

## 2. Materials and Methods

### 2.1. Materials

In this study, recycled polypropylene (PP-r) Eco Meplen IC M10 BK (0.94 g/cm^3^, 10 g/10 min: MFI) supplied by MEPOL S.r. (Treviso, Italy) (was chosen as the matrix for the functionalised wood fibre nanocellulose composites (nW-PPr). The WF was sourced locally as a by-product of plywood grinding and consisted of spruce and pine wood (about 20–80 wt%), as reported here [49,51]. The chosen nanocellulose fibres (nC) for the nW-PPr composites were purchased from MAIN University (Philadelphia, PA, USA) (with PDI~0.81. Ultrapure water was provided by a MilliQ water purifier (Millipore Corporation, St. Louis, MO, USA). The electrolyte solutions required for surface zeta potential analysis were prepared from KCl p.a. (Wilkes-Barre, PA, USA), 0.051 mol/L HCl, and 0.051 mol/L KOH purchased from Carl Roth, Baden-Wuerttemberg, Germany.

### 2.2. Preparation of the nW-PPr Composites

The nW-PPr composites reinforced with WF functionalised with nC were prepared with constant 50 wt% mass fractions of WF and three different individual nC contents, 1.0, 3.0, and 9.0 wt%, with respect to the entire composite (Table 1). In total, three composites’ materials with PP-r were produced in this way: Dried WF (50 wt%) were mixed in a flask with a mechanical stirrer with a nanocellulose nC suspension with three different contents of nC (1.0, 3.0, and 9.0 wt%). After that, the functionalised WF were dried 3 h in a ventilated oven at 110 °C.

Further, recycled polypropylene-based composites reinforced with functionalised wood fibres by cellulose nanofibres (nW) were prepared by the compounding extrusion method. The PP-r and dried nW were mixed and fed to the feed unit of a twin-screw extruder, PolyLab HAAKE Rheomex PTW 16, Thermo Haake (Karlsruhe, Germany). To ensure uniform distribution of WF in the polymer matrix, all the materials were extruded three times. After each extrusion, the material was pelletised by a pelletiser, and the material was fed to the feeder again.

The samples under investigation are described in Table 1.

### 2.3. Characterisation Methods

X-ray diffractometry (XRD) was employed to evaluate the effect of nW on the semicrystalline structure of all the prepared recycled-based composites at RT. The XRD spectra were taken using a MiniFlex II XRD system (Rigaku Co., Takatsuki city, Japan) using a Cu K α radiation source at 40 kV. The X-ray diffraction patterns were recorded for the angles in the range of 2θ, with a scanning step of 10 °C/min (λ = 0.154) and measured from 10 to 60°. The spectra were fitted using the crystallographic program Topas2R 2000 (Bruker AXS, Madison, WI, USA) based on a convolution approach (Pawley method).

The attenuated total reflectance Fourier transform infrared spectroscopy (ATR-FTIR) spectra were monitored on a Perkin Elmer Spectrum (Waltham, MA, USA). The spectra were recorded in the wavenumber range from 600 to 4000 cm^−1^ at room temperature (RT). Each spectrum was determined as an average of 32 scans at a resolution of 4 cm^−1^ for measuring the background. All the spectra presented here were baseline-corrected and smoothed after the measurement.

Thermogravimetric analysis (TGA) of the nW-PPr composites was carried out using a TGA/DSC 3+ (Mettler Toledo, Greifensee, Switzerland) in 40 µL aluminium crucibles. The samples of the composites (6–10 mg) were placed in alumina crucibles, while an empty alumina crucible was used as a reference and heated from 25 to 800 °C in a 50 mL/min flow of N_2_ at heating rates of 10 °C/min. Continuous recordings were taken of sample temperature, sample mass, its first derivative, and heat flow.

Tuning the structural properties of the nW-PPr composites enhanced the changes in their thermal properties, too. A differential scanning calorimeter, DSC3, Mettler Toledo, was used for a detailed investigation of the nW-PPr composites’ crystallisation, melting behaviour, and nucleation efficiency. About 10 mg samples were calibrated in standard aluminium pans. The heat–cool–heat temperature scan was performed with a heating/cooling rate of 10 °C/min in an N_2_ atmosphere (20 mL/min). 

A first heating scan, up to 200 °C, was performed to erase the thermal history, and, subsequently, main thermal protocols were employed as follows: The non-isothermal crystallisation kinetics of these composites were investigated by conducting cooling scans at rates of 2.5, 5.0, 10.0, 15.0, 20.0, and 30.0 °C /min from 200 to 25 °C. Prior to the heating or cooling scan, the samples were held at 200 °C for 5 min under a nitrogen atmosphere to erase the thermal history and prevent self-seeding of the PP-r.

The degree of crystallinity (χ) and melting temperature (T_m_) were determined following Equation (1):

(1) $$\chi =\frac{∆H_{m}}{∆H_{m}^{0}\cdot (1-W_{wt\%})}\times 100$$

where χ is the degree of crystallinity, ∆H_m_ is the melting enthalpy, ∆H_m_^0^ is the melting enthalpy of the 100% crystalline PP, and is 207 J/g [52]. W (wt%) is the mass fraction of wood nanofibres in the composite’s material.

The morphological characteristics of the nW-PPr composites were investigated employing scanning electron microscopy (SEM). Specifically, a Carl Zeiss FE-SEM SUPRA 35 VP (Jena, Germany) equipped with a GEMINI field emission module was employed for this analysis. To facilitate imaging, the prepared plastic composites were affixed to double-sided adhesive conductive carbon tape and secured onto an aluminium specimen holder. The holders containing the affixed samples were positioned within the microscope and subjected to imaging under controlled conditions: an accelerating voltage of 1 keV, a 30 µm aperture, and an adjustable working distance to optimise the focus. The imaging encompassed both low magnification (700×) and high magnification (5000×) settings. The acquisition of SEM images was executed in secondary electron mode (SE), utilising an in-lens detector to enhance the resolution. This approach ensured a detailed exploration of the morphological features of the functionalised nW-PPr composites at varying scales.

The measurement procedure was executed with 1 mM KCl, and the streaming potential was gauged using an Ag/AgCl electrode. Solutions of 0.05 M HCl and KOH were employed for pH modulation throughout the scanning measurement concerning pH. It is noteworthy that the electrolyte solution underwent continuous purging with N_2_ gas during the entire measurement process, ensuring an environment conducive to accurate zeta potential determination.

The surface zeta potential was measured by streaming potential on a SurPASS 3 instrument (Anton Paar GmbH, Graz, Austria). The adjustable gap cell was used to determine the surface zeta potential of untreated PPr, and PPr was modified with functionalised WF, while a cylindrical cell was used for the functionalised WF. Two pairs of identical specimens, approximately 20 mm × 10 mm in size, were cut out and adhered to sample holders with double-sided adhesive tape. The distance between the opposing samples was adjusted to (110 ± 10) µm during the rising process using a 1 mM KCl electrolyte solution using an adjustment knob. The measurement was performed with 1 mM KCl, and the streaming potential was measured with the Ag/AgCl electrode. During the scan measurement, as a function of pH, 0.05 M HCl and KOH were used for pH adjustment. During the performed measurement, the electrolyte solution was purged continuously with N_2_ gas.

The dynamic mechanical properties were measured using a DMA 8000 (Perkin Elmer, Waltham, MA, USA) dynamic mechanical analyser. All the composites and neat PP-r were tested from tensile test specimens. The samples were studied in flexure using a dual cantilever beam support. The distance between the supports was 12.6 mm, the frequency was 1 Hz, and the amplitude was 0.005 mm. The temperature range of the measurement composites was from 26 °C to 180 °C, with a heating rate of 1 K/min.

## 3. Results and Discussion

The study used XRD to examine how the crystallographic structure of the nW-PPr composites changed with the addition of the nW coupling agent. Non-functionalised WF is recognised to act as a nucleating agent, enhancing interfacial interaction, crystallographic structure, and morphology. Nucleating agents play an important role in the extrusion of polymers. They act as templates, encouraging the formation of small crystalline regions within the polymer matrix, which results in improved mechanical properties, such as stiffness, tensile strength, and impact resistance [53]. Among the various crystalline modifications, the monoclinic α-phase is a prevalent structure in polypropylene [18,46,47,48,49]. The existing literature indicates that transcrystallisation occurs around the WF, affecting the interfacial adhesion, load transfer, and (thermo) mechanical properties. However, our XRD analysis of the nW-PPr composites did not show evidence of transcrystallisation; instead, the predominant observation was the α-phase of the polypropylene [50,51,52].

**Figure 1:**
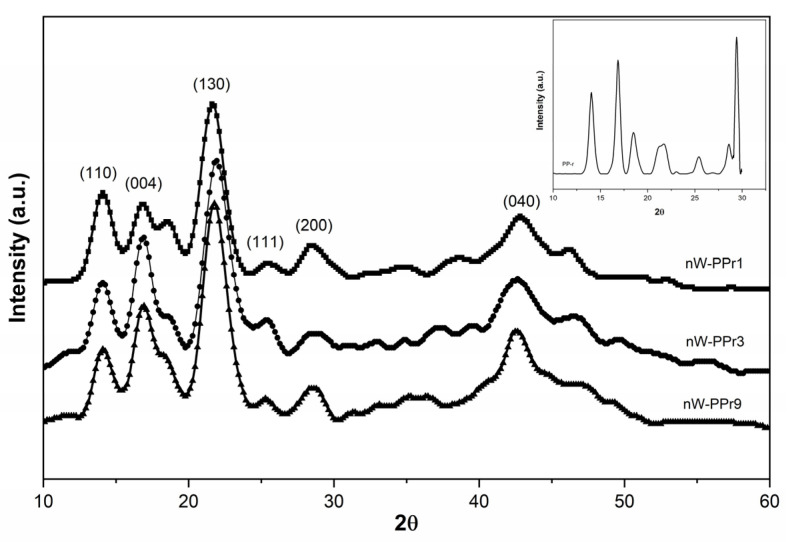
X-ray diffraction (XRD) diffraction pattern of the PP-r polymer and nW-PPr composites.

The crystallographic structures of nW-PPr as the results of nW interaction with the nW-PPr composites were measured by XRD measurements. Figure 1 represents the XRD diffraction patterns in the diffractogram in the 2θ range of all the nW-PPr composites, varying at 1.0 wt%, 3.0 wt%, 9.0 wt% nC content, and neat PP-r. The PP-r (in the inset) and nW-PPr composites (Figure 1) diffraction peaks displayed well-defined peaks, typical of a semicrystalline structure, and positioned at 2θ angles of 13.9° (110), 16.8° (004), 18.5° (130), 21.4° (111), and 28.6° (200). Generally, it was observed clearly that there were no changes in tacticity within the crystallographic structure of the composites based on recycled PP. All the peaks reflected the α-phase of polypropylene, as supported by the literature [54,55,56,57].

**Figure 2:**
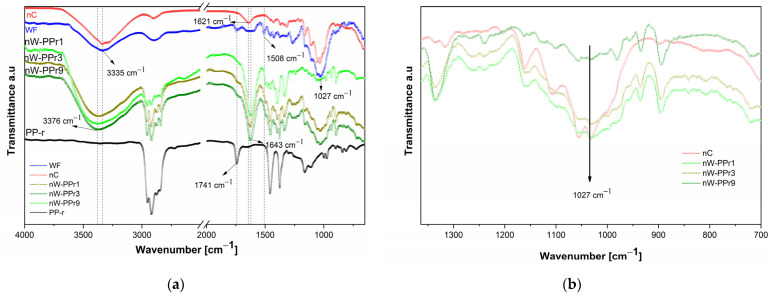
(**a**) The comparative ATR-FTIR spectra of nC, WF, PP-r, and nW-based PP-r composites over the large wavenumber range. (**b**) Dependence on the intensity of the peak at 1027 cm^−1^ on the nW addition content representing the stretching vibration of the C-O and C-O-C groups of the nW-PPr composites.

The ATR-FTIR was used to investigate the dependence of the incorporation of nW filler in a PP-r polymer on the chemical structure of the nW-PPr composites. The results of the ATR-FTIR analysis are given in Figure 2. The neat PP-r polymer, nW-PPr1, nW-PPr3, and nW-PPr9 composites were monitored additionally by measuring the ATR-FTIR spectra. The PP-r polymer showed a band at 2917 cm^−1^ (CH_2_ asymmetric stretching), 2951 cm^−1^ (CH_3_ stretching), 2868 cm^−1^ (CH_2_ asymmetric stretching), 1456 cm^−1^ and 1374 cm^−1^ (CH_3_, CH_2_ rocking vibration). Additionally, it was observed that the characteristic peak at 1741 cm^−1,^ assigned to the C=O (OH) stretching vibration of a carbonyl group, indicated the presence of a carbonyl group of an additive oxygen process. Generally, it is known that the carbonyl groups (ketone, aldehyde, carboxyl, ester, and hydroxyl groups) are formed during the oxidation of polyolefin in the process of extrusion (thermo-oxidation and photo-oxidation) and depend on the chemical and physical structure. Moreover, the formation of these carbonyl groups can act as a coupling agent, increasing compatibility in recycled polymer composites (the use of recycled plastic in WF composites) [49,58]. The bands at 3400 cm^−1^ (O-H stretching), 2950 cm^−1^ (the asymmetric and symmetric methyl and methylene stretching groups), 1741 cm^−1^ (C=O stretching vibration in lignin and hemicellulose), 1505 cm^−1^ and 1270 cm^−1^ (C=C, C-O stretching or bending vibrations of the groups in the lignin), 1452 cm^−1^, 1422 cm^−1^, 1374 cm^−1^ (C-H, C-O deformation, bending or stretching vibrations of the lignin groups and carbohydrates), 1165 cm^−1^, 1050 cm^−1^, and 1030 cm^−1^ (C=O, C-H, C-O-C, C-O deformations or the stretching vibrations of different groups in carbohydrates and the glycosidic linkage) indicated the presence of typical functional groups of WF. The nC exhibited peaks at 3335 cm^−1^ (O-H stretching) and 2950 cm^−1^ (symmetric and asymmetric C-H stretching), and the region between 1000 and 1070 cm^−1^ is the region of alcohols (~1030 cm^−1^) and is assigned to the stretching vibrations of different groups in carbohydrates. Further, the peak at 1641 cm^−1^ illustrates the vibrations of the remaining absorbed water or OH vibration of the alcohol group. The spectra of the nW-PPr composites with the addition of 1.0, 3.0, and 9.0 wt% of nW in the PP-r polymer are shown in Figure 2a, and the main bands that were observed were at 3376 cm^−1^ (O-H stretching), 1505 cm^−1^, 1270 cm^−1^ (C=C, C-O stretching or bending vibrations of the groups in the lignin), 1452 cm^−1^, 1422 cm^−1^, 1374 cm^−1^ (C-H, C-O deformation, bending or stretching vibrations of the lignin groups and carbohydrates), 1643 cm^−1^ (O-H stretching, vibration of the remaining adsorbed water), and 1027 cm^−1^ (alcohols, and assigned to the stretching vibrations of different groups in the carbohydrates) [9,59]. 

**Figure 3:**
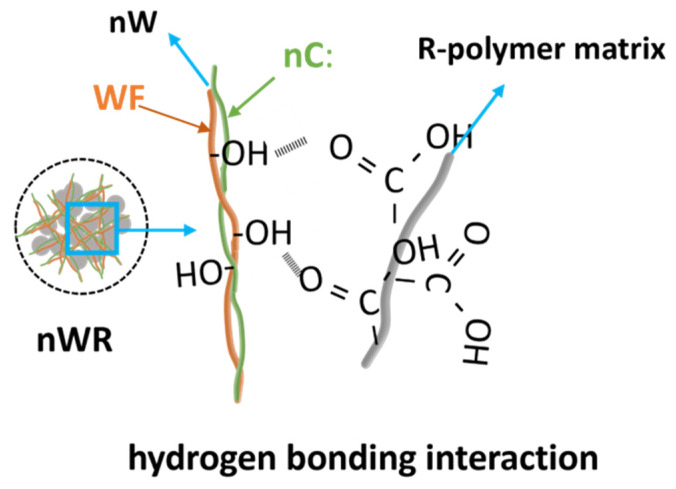
The proposed structure of the nW-PPr composites.

Interestingly, in the infrared spectra of all three nW-PPr composites (i.e., nW-PPr1, nW-PPr3, and nW-PPr9), the alteration was observed of the peaks at 3376 cm^−1^ and at 1741 cm^−1^. The shifting to the highest wavenumber of the pronounced band at 3376 cm^−1^ that represented O-H stretching vibration and a disappearing band at 1741 cm^−1^ of the carbonyl group can be associated with the formation of an H-bond between the nW and PP-r in the nW-PPr polymer composites (as proposed schematically in Figure 3). In fact, it was also seen that the intensity of vibration bands determined at 1027 cm^−1^ (typical for different groups in carbohydrates) for the nW-PPr polymer composites’ structure increased with the increasing addition of functionalised WF, as shown in the spectra in Figure 2b.

**Figure 4:**
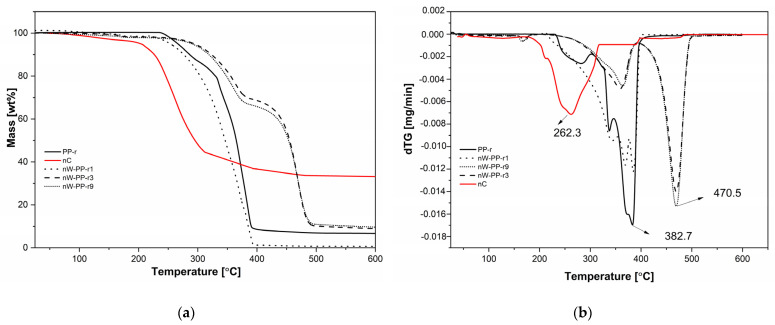
(**a**) TGA profile/thermograms, and (**b**) dTG curves of neat PP-r, nC, and PPr filled composites with nW.

Generally, thermogravimetric analysis of the nW-PPr composites was carried out to assess their thermal stability and degradation temperature. The thermal degradation of PP-r, nC, and nW-PPr filled with 3 nW content was measured by TGA analysis. Figure 4 represents the TGA thermograms and derivative mass loss (dTG) curves of PP-r, nC, and all three nW-PPr composite samples. For neat PP-r, the degradation temperature is 264 °C, whereas the final degradation temperature is 382 °C. The small weight loss of temperature below 100 °C of nC (Figure 4) was observed due to the water evaporation and the second degradation step at 262 °C. Additionally, the residual mass of nC reached ~37% at 600 °C, indicating that nC does not degrade at this specific temperature. Furthermore, Figure 4 shows that the addition of functionalised nW fibres into the PP-r matrix enhanced the thermal stability of the composites, and it is also clear that the main peak of degradation was related to the degradation of nC and the WF component. The thermal degradation profile and thermal stability of the nW-PPr1 composites with the lowest content of added nC to WF (1.0 wt%), represented in Figure 4, was almost similar to the PP-r polymer. In particular, the first degradation temperature was seen at 264 °C, whereas the final degradation temperature was similar to neat PP-r, i.e. 382 °C. For the nW-PPr composites with a larger addition of functionalised nW (3.0 wt% or 9.0 wt%), the thermal profile was changed in comparison with the neat PP-r and nW-PPr1 composites. It seems that the composites nW-PPr3 and nW-PPr9 resulted in better thermal stability compared to the neat PP-r polymer. Furthermore, the peak shift of degradation temperature at 264 °C altered to a higher value of temperature. The degradation profile represented the first degradation step at 382 °C and the second at 470 °C. Hence, using functionalised nW in the PP-r polymer enhanced the thermal profile stability of the composites, and, with the addition of 3.0 wt% and 9.0 wt% nW, the thermal degradation shifted to a higher temperature. Based on the thermograms shown, it is evident that the composites nW-PPr3 and nW-PPr9 are more stable compared to nW-PPr1 and neat PP-r. The maximum rate of the main decomposition step of nW-PPr1 and neat PP-r is shown at 382 °C, while the composites nW-PPr3 and nW-PPr9 at 470 °C (Figure 4b). This may also be related to the formation of hydrogen bonds between the nW and PP-r in the nW-PPr that can enhance the thermal stability of materials because they create a stronger interaction between the molecules (Figure 3) that can enhance thermal stability by influencing interfacial stability, molecular structure, and the maintenance of structural integrity, thereby contributing to the overall heat resistance of the material, and improving the overall thermal stability of the polymer and filler system [60]. 

The changes in the thermal profile of all three nW-PPr composites in comparison with neat PP-r could be explained by functionalisation within the nW in the PP-r, which enhanced the interaction between the filler and PP-r matrix and, in this way, also provided better dispersion of the nW filler in the composites. Similarly, this kind of thermal increase was previously reported in thermoplastic composites with alkaline peroxide-pretreated WF. These results suggest that the addition of functionalised WF to PP-r could serve as an alternative to prime neat, recycled polymer.

**Figure 5:**
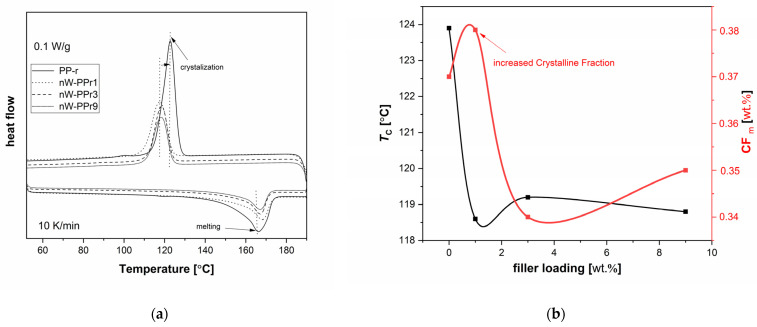
(**a**) DSC curves by scan 2—heating and cooling at 10 K/min upon erasing the thermal history. The heat flow has been adjusted to the samples’ masses. The vertical dash-dotted lines represent the shifted temperature positions of the nW-PPr composites’ thermal point regarding neat PP-r. (**b**) Filler loading effect on T_c_ estimated from melting.

In order to explain further the thermal properties related to the effect of nW fillers on the thermal properties of nW-PPr composites, and, in particular, on the changes in crystalline structure and crystallisation process that relies on the interfacial interaction, differential scanning calorimetry (DSC) is used in this research. All the represented thermal parameters in the thermal analysis are listed in Table 2. The results were evaluated in terms of thermal properties, namely, T_m_ is the melting temperature, ΔH_m_ is the melting enthalpy, T_c_ is the temperature of crystallinity, and Xc is the degree of crystallinity. The thermograms represented in Figure 5 show that, during the heating (Figure 5a), neat PP-r exhibited a T_m_ of 166.8 °C and, during the cooling, a T_c_ of 123.9 °C. The presence of nW in the nW-PPr composites with different varying addition of content resulted in enhanced thermal properties. Namely, the results indicated that the melting temperature and crystallisation temperature are complex and did not change significantly with increasing the amount of nW, which was determined at around 166 °C. On the other hand, the nW changed the crystallisation process of the nW-PPr composites compared to the neat PP-r composites, resulting in variable degrees of crystallinity. Thus, the degree of crystallinity of the nW-PPr composites with the 1.0 wt% of functionalised nW exhibited the highest crystallinity, approximately 40.1%, compared to neat PP-r with 37.9%. The degree of crystallinity decreased with the further addition of nW. For the nW-PPr3-based composites, the degree of crystallinity decreased further from 40% to 35% and represented a 5% decrease.

Additionally, the melt crystallisation was recorded for all composites (Table 2 and Figure 5a). The results obtained via T_c_ in Figure 5a and the estimated crystalline fraction from melting, CF_m_, are presented in Figure 5b. Employing the melting enthalpy, ΔH_m_, normalised to the PP-r fraction, and comparing with the heat of fusion of fully crystalline PP-r, ΔH_PP_, 100% = 207 J/g, the estimated value of CF_m_ was calculated according to Equation (2).

(2) $${CF}_{m}=\frac{{∆H}_{m}}{w_{PP-r\cdot }{∆H}_{PP-r,100\%}}=\frac{{∆H}_{m,n}}{{∆H}_{PP-r,100\%}}$$

The results showed that the filler plays an important role in the nucleation process of PP-r-based composites. On the one hand, it relates to more pronounced interfacial interaction between the polymer and filler in the case of the composites nW-PPr3 and nW-PPr9 that decreased the CF_m_, and, on the other hand, the smallest amount of addition of nW to PP-r played a significant role in the nucleation of PP-r, which resulted in increased crystallinity and CF_m_ (Figure 5b). These effects suggest quite strong interfacial interactions, as obtained from our previous observation by ATR-FTIR (Figure 2a). Also, it is clearly known from the literature for other polymer composites that the nucleating process is connected with absent interfacial interaction and also with a slight increase in the polymer free volume [61] in the case of composites. 

**Figure 6:**
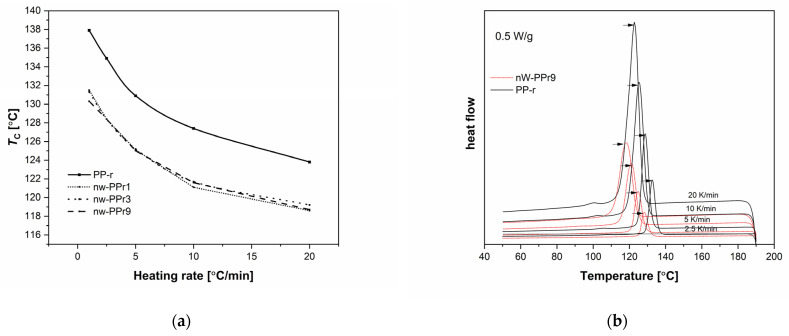
DSC thermograms of non-isothermal crystallisation of PP-r polymer and composites nW-PPr1, nW-PPr3, and nW-PPr9 composites at various cooling rates: (**a**) The temperature of crystallisation dependence on heating rate (2.5, 5.0, 10.0, and 20.0) of neat PP-r and nW-PPr1, nW-PPr3, and nW-PPr9. (**b**) The comparison of DSC curves by crystallisation and at the different cooling rates for the samples PP-r and composites nW-PPr9. The heat flow values have been normalised to the mass of each sample.

To go into more detail, the latter effects are examined in the following by non-isothermal crystallisation kinetics analysis (Figure 5 and Figure 6). The following Figure 6 shows the results measured with DSC and shows how the polymer crystallised at a high temperature, consequently, with high polymer chains’ mobility but having varying supercooling. In this way, the nucleating role of the nW fillers can be explained more directly. Therefore, Figure 6a explains the typical DSC curves of heat flow as a function of temperature at different cooling rates for neat PP-r and nW-PPr1, nW-PPr3, and nW-PPr9 composites. The results shown in Figure 6 indicate that the crystallisation temperature of neat PP-r changes with the cooling rate upon the addition of nW fibre. As the heating rate decreases, the temperature of crystallinity decreases and shifts to the lowest value of temperature. With a heating rate of 2.5 °C/min, the temperature of crystallisation for PP-r was 138 °C, while, for all nW-PPr, nW-PPr3, and nW-PPr9 composites, it was around 131 °C. Also, in Figure 6b, from the time evolution of crystallisation of the composites and neat PP-r polymer, it is quite clear that crystallisation was postponed in the composites rather than in the neat PP-r. The temperature of crystallinity was affected by the addition of nW. Compared to neat PP-r, the Tc of nW-PPr9 measured at different cooling rates of the composite was 5 °C lower, i.e., at 118 °C, which represented about a 6% decrease in Tc when the highest amount of nW was added to the neat PP-r. A similar shift to the lower value in crystallisation temperature was also obtained for the nW-PPr9 composites. At 2.5 °C/min, the temperature of crystallisation for PP-r decreased from 133 °C to 128 °C for the nW-PPr9 composite material. In this case, the difference was also about 5%.

**Figure 7:**
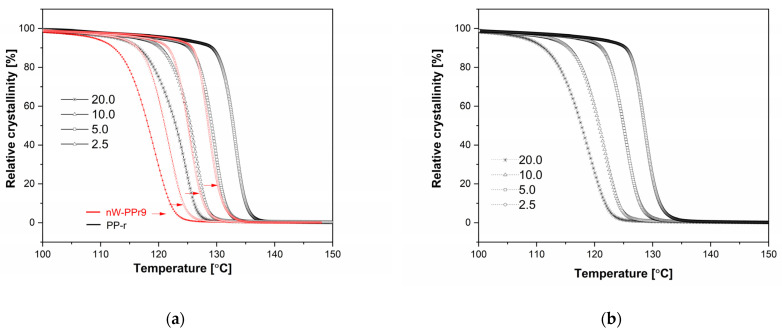
Plots of X(t) versus T for samples: (**a**) PP-r comparing with nW-PPr9; (**b**) nW-PPr1.

These results are in line with the results of the degree of crystallinity (Table 2), as they show that the addition of nW affects the crystallisation process of PP-r-based composites.

(3) $$X\left(t\right)=\frac{\int_{T0}^{T}\left(\frac{dHc}{dT}\right)dT}{\int_{T0}^{T\infty }\left(\frac{dHc}{dT}\right)dT}$$

where T_0_ and T are the onset and end temperatures of crystallisation, respectively, and H_c_ is the enthalpy of crystallisation. According to the time–temperature transformation Equation (3) [62], in which T is the temperature at crystallisation time t, and u is the cooling rate. Plots of X(t) versus T or t for the samples crystallised at different cooling rates are shown in Figure 7. The half-time of crystallisation (t1/2), that is, the time taken for 50% of the total crystallisation to occur, was used to characterise the crystallisation rate. Here, the results presented in Figure 7a,b and obtained from Equation (3) revealed that the t1/2 of PP-r and its composites decreased with increasing cooling rates. The results also indicated that all the prepared nW-PPr composite materials exhibited enhanced effects on relative crystallinity. Namely, the composites with 1.0 wt% addition of nW consequently improved the nucleation ability with improved percent of crystallinity and crystallisation process of polymer composites, as was explained previously, and, the highest amount of nW filler postponed the crystallisation process of polymer composites, as was explained in the Figure 7a. and also proved by the results in Table 2.

**Figure 8:**
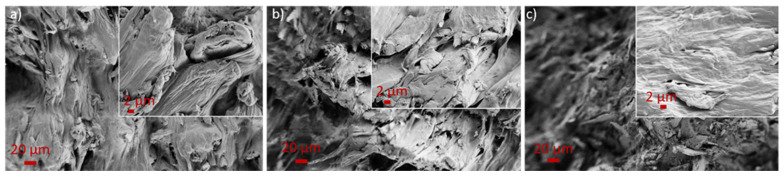
SEM images of (**a**) nW-PPr1, (**b**) nW-PPr3, and (**c**) nW-PPr9.

To elucidate the impact of incorporating nW into PP-r on its morphological characteristics, a scanning electron microscopy (SEM) analysis of the functionalised PP-r samples was conducted, as depicted in Figure 8. The lower magnification images (700×) provide a representative overview of the composites, while the higher magnification (5000×) affords a more detailed insight into the nW and their interaction with the polymer matrix (refer to the insets). The SEM images revealed that individual functionalised nW was integrated within the PP-r polymer matrix. Notably, when WF were functionalised with 1.0 and 3.0 wt% nC (Figure 7a,b), the individual nW entities became distinctly visible, and the PP-r adhered to them. Conversely, with a higher concentration of nC used for WF modification, the individual nW entities were less perceptible, and they were predominantly obscured by the polymer matrix (Figure 8c).

**Figure 9:**
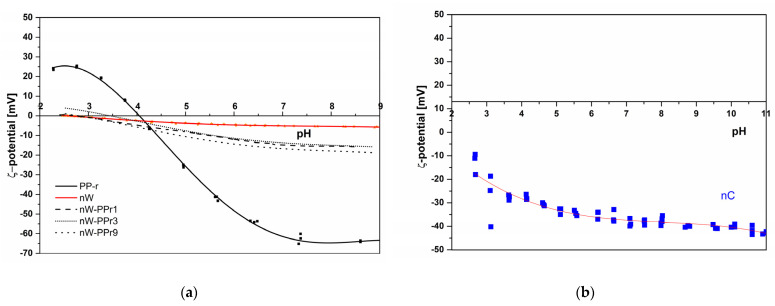
(**a**) Surface zeta potential as a function of pH for the nW, PP-r, and composite materials. The zeta potential for neat nC is shown (**b**) for comparison purposes.

Zeta potential as a function of pH is presented in Figure 9. It provides information about the surface charge and its variations with pH. Zeta potential is a crucial parameter for understanding the surface charging behaviour of solid materials. Polypropylene (PP) is a plastic material whose charge can be influenced by the presence of functional groups on the surface or adsorbed ions. Our curve is typical for neat PP, with an isoelectric point at pH 4. Below pH 4, it exhibited a positive zeta potential, and above pH 4, a negative zeta potential. This behaviour is characteristic of a polymer or another hydrophobic material surface lacking surface functional groups, and it results from the specific adsorption of ions from the surrounding liquid medium. At pH levels below the isoelectric point, the specific adsorption of H_3_O^+^ ions predominated, imparting a positive interfacial charge, while, above pH 4, the negative zeta potential was due primarily to the adsorbed OH^−^ ions on the surface [63]. The PP showed a relatively high negative zeta potential plateau (at −65 mV), which resulted from adsorbed OH^-^ ions, as well as the hydrophobic nature of PP. After integrating WF and nC into the PP-r, the isoelectric point shifted to a lower pH value, similar to that of wood or nanocellulose itself (Figure 9b). This demonstrates clearly the availability of both fillers on the PP-r surface. The shift of the isoelectric point to a more acidic environment indicates an increase in material acidity due to the introduction of more carboxylic acidic groups and other acidic compounds. 

With the increased addition of nC mass, we can also observe clearly a greater increase in acidity (the value of pH for nW-PPr varied from 3.4 to 2.6 and 2.7) as proof of the nanocellulose functional groups’ presence and/or the availability of (COOH and OH).

The negative zeta potential plateau values after attaching WF and nC into PP-r should be even lower due to increased polarity and the introduction of acidic groups. However, an increase to the less negative zeta potential may be observed, which is connected to a decrease in hydrophobicity and converting material into hydrophilic, resulting in a sharper shift of negative zeta potential plateau levels to less negative values (from −65 mV to an interval of −15 to −12 mV for samples with added WF and nC).

Controlling the surface charge of PP-r or any of its modifications holds great significance, as it impacts its interactions with other materials in various industrial and research settings. It is particularly important for applications such as packaging, where surface charge is important for applicable properties, such as microbial inhibition, wettability, antioxidant activities, etc.

**Figure 10:**
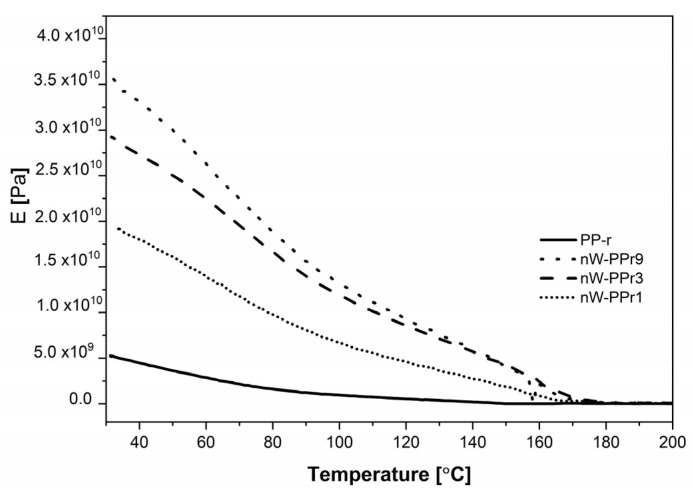
DMA curves of PP-r and nW-PPr1, nW-PPr3, and nW-PPr9 composites as a function of temperature.

The results obtained using dynamic mechanical analysis (DMA) as a powerful tool to study the viscoelastic behaviour of polymers and composites are shown in the figure below (Figure 10). The effect of nW content on the dynamic mechanical properties of the composites was determined. The results of the DMA are presented as complex modulus and storage modulus (E′) and define the measure of the elastic behaviour of the sample of PP-r and nW-PPr composites in the temperature range of 10 to 200 °C. In a DMA test, the storage modulus (E′) measures the energy stored in the specimen to define the structure of the material in terms of parameters such as molecular weight and cross-linking/interfacial interaction for polymers or can be used to explain the response of a material to environmental or external variables such as temperature, time or frequency, and relative humidity [64,65].

All the samples of nW-PPr composites based on recycled polymer PP-r showed relatively similar dynamic mechanical behaviour with observed minor changes, as shown in Figure 10. The storage modulus values of all three composites were very close over the whole temperature range. The higher E′ value measured over the whole temperature range compared to neat PP-r was observed for composites with the highest addition of functionalised nW (9.0 wt%), namely, the nW-PP-r9 composites. The storage modulus value for all the composites continued to follow the trend: nW-PPr9 ≥ nW-PPr3 ≥ nW-PPr1 ≥ PP-r. Thus, the nW-PP-r composites exhibited an increase in E′ compared to the neat PP-r over the observed temperature range. However, the storage moduli of the nW-PP-r9 (3.5·10^10^ Pa) composites were slightly higher than those of the nW-PPr3 (2.9·10^10^ Pa) and nW-PPr1(1.8·10^10^ Pa). The alteration of the storage moduli of the nW-PPr composite could be explained by the following: (i) the changes in the interaction between the functionalised fibre nW and polymer compared to the neat PP-r polymer; (ii) the changes in the structure of the nW-PPr composites; and (iii) the interfacial adhesion with the polymer as it was found previously in the literature [65,66]. As was explained before with ATR-FTIR, DSC, in our investigation, the interfacial interaction that provided changes in the structure of nW-PP-r between nW and the PP-r matrix was probably the main determinant in the increased value of the storage moduli. In addition, the results proved the prediction stated in the literature on the main properties affecting the storage modulus, including the morphology of the system, the intrinsic properties of the components, and the interfacial nature between the phases, which mainly governed the dynamic mechanical properties of the composite. To concretise, the increasing content of nW in the polymer composites increased due to the better interfacial interaction between the PP-r and nW, which resulted in good adhesion, and to a higher value of storage modulus of the composites.

Even more, the storage moduli of the composites were observably higher than the neat PP-r, which was an indication of interaction between the filler and polymer, leading to enhanced crystallinity and morphology with good adhesion, which resulted in the increased storage moduli.

## 4. Conclusions

The investigation of functionalised WF-reinforced recycled plastic composites based on thermoplastic PP polymers was prepared as high-added value composites following the Green Deal strategy. Prior to the study of the main role of the coupling agent in the final properties in terms of their structural properties, we examined here how the functionalised WF enhanced the structural changes, and the crystallisation process of r-PP provided improved surface and mechanical changes. 

The crystallographic structures of all three composites nW-PPr showed the presence of an α-phase of polypropylene. In detail, the coupling agent of functionalised WF provided the formation of an H-bond between the nW and PP-r in the nW-PPr polymer composites and affected the thermal properties as well. Thus, the addition of functionalised WF, by the role of coupling agent either in 1.0 wt% acting as a nucleating agent, or, on the other hand, in 3.0 wt% and 9.0 wt%, showed the strongly suppressed crystallisation process no more. The effects were explained mainly by the more pronounced interfacial interaction between the polymer and filler in the case of nW-PPr3 and nW-PPr9 composites. The smallest amount of addition of nW to PP-r played a significant role in the nucleation of PP-r, which resulted in increased crystallinity and a weak polymer-filler interaction also by the role of coupling agent in the postponed crystallisation temperature. On the other hand, the structural changes in composites could also have a strong relationship with surface properties such as morphology and zeta potential. Stronger effects on the surface morphology of functionalised WF were observed with 1.0 and 3.0%, with a more evident presence of nW. Conversely, with a larger amount of nW, no visible filler was detected on the surface. The pronounced effect in the structure of composites compared to neat r-PP were found to enhance the zeta potential, suggesting improved interactions between the filler and polymer. The lower isoelectric point of the composites compared to neat r-PP demonstrates the availability and distribution of fillers on the r-PP surface. This could be correlated with an increase in the acidity of the material, due to the introduction of more carboxylic acid groups and other acidic compounds. Finally, the correlation of the role of chemical structure and interfacial adhesion with the polymer of all three composites resulted in dynamic mechanical analysis with higher storage moduli of composites than neat r-PP, indicating interaction between the filler and polymer leading to increased crystallinity and morphology, with good adhesion that resulted in the increased storage moduli. The results of this study highlight the use of environmentally friendly components in commercially available polymers, which have improved properties and opened up possibilities for various applications, such as packaging.

## Figures and Tables

**Table 1:** Sample description and naming of the composites under study.

Sample Description	Name of Sample
Recycled polypropylene	PP-r
PP-r + 50%WF + 1.0 wt% nC	nW-PPr1
PP-r + 50%WF + 3.0 wt% nC	nW-PPr3
PP-r + 50%WF + 9.0 wt% nC	nW-PPr9

**Table 2:** Thermal parameters of the investigated composites: temperature of crystallisation (T_c_), crystallisation enthalpy (ΔH_C_), T_m_ is the melting temperature, and Xc is the degree of crystallinity crystalline fraction estimated from the melting.

Sample	T_c_ (°C)	ΔH_C_ (J/g)	T_m_ (°C)	χ (wt%)
PP-r	123.9	80.63	166.8	37.9
+ 50%WF+ 1.0 wt% nC	118.6	44.7	167.7	40.1
+ 50%WF+ 3.0 wt% nC	119.2	36.1	166.6	34.7
+ 50%WF+ 9.0 wt% nC	118.8	31.6	166.7	35.6

## Data Availability

Data is contained within the article.
